# Effect of Surface Structure Complexity on Interfacial Droplet Behavior of Superhydrophobic Titanium Surfaces for Robust Dropwise Condensation

**DOI:** 10.3390/ma14154107

**Published:** 2021-07-23

**Authors:** Je-Un Jeong, Dae-Yun Ji, Kwon-Yeong Lee, Woonbong Hwang, Chang-Hun Lee, Sung-Jae Kim, Jeong-Won Lee

**Affiliations:** 1 Department of Mechanical Engineering, Chosun University, Gwangju 61452, Korea; jjw4648@chosun.kr; 2 Department of Mechanical and Control Engineering, Handong Global University, Pohang 37554, Korea; wleodbs12@naver.com (D.-Y.J.); kylee@handong.edu (K.-Y.L.); 3 Department of Mechanical Engineering, Pohang University of Science and Technology (POSTECH), Pohang 37673, Korea; whwang@postech.ac.kr; 4 Department of New Biology, Daegu Gyeongbuk Institute of Science and Technology, Daegu 42988, Korea; leech@dgist.ac.kr; 5 Department of Orthopaedic Surgery, Dongtan Sacred Hospital, Hallym University, Hwasung 18450, Korea; sung1383@hanmail.net

**Keywords:** superhydrophobic surface, condensation heat transfer, micro-nanostructure, droplet behavior, dropwise condensation

## Abstract

In general, the dropwise condensation supported by superhydrophobic surfaces results in enhanced heat transfer relative to condensation on normal surfaces. However, in supersaturated environments that exceed a certain supersaturation threshold, moisture penetrates the surface structures and results in attached condensation, which reduces the condensation heat transfer efficiency. Therefore, when designing superhydrophobic surfaces for condensers, the surface structure must be resistant to attached condensation in supersaturated conditions. The gap size and complexity of the micro/nanoscale surface structure are the main factors that can be controlled to maintain water repellency in supersaturated environments. In this study, the condensation heat exchange performance was characterized for three different superhydrophobic titanium surface structures via droplet behavior (DB) mapping to evaluate their suitability for power plant condensers. In addition, it was demonstrated that increasing the surface structure complexity increases the versatility of the titanium surfaces by extending the window for improved heat exchange performance. This study demonstrates the usefulness of DB mapping for evaluating the performance of superhydrophobic surfaces regarding their applicability for industrial condenser systems.

## 1. Introduction

Humans often turn to nature for inspiration when developing new technologies. The lotus leaf, which provided a template for researchers studying superhydrophobic surfaces, is a classic example and has been referenced in many applied studies on superhydrophobicity [1,2,3,4,5,6,7]. The superhydrophobic surface property is determined by the contact angle. When a droplet touches a solid surface, the surface tension is balanced between the liquid, the solid, and the gas and the angle between the solid and the liquid surface is defined as the contact angle. This theory is described by Young’s equation:

(1) $$\cos\theta =\frac{\gamma_{\mathrm{SG}}-\gamma_{\mathrm{SL}}}{\gamma_{\mathrm{LG}}},$$

where *θ* is Young’s contact angle and $\gamma_{\mathrm{SG}}$*,* $\gamma_{\mathrm{SL}}$*,* and $\gamma_{\mathrm{LG}}$ are the surface tension between the solid-gas, solid-liquid, and liquid-gas interface, respectively. Depending on the surface energy of the solid, Young’s contact angle can be changed. In addition, if the contract angle is smaller than the right angle, it is classified as hydrophilic. On the other hand, if it is larger than the right angle, it is classified as hydrophobic. Physical surface modification can maximize the original hydrophilicity/hydrophobicity by providing micro/nano structures. When the contact angle is more than 150°, it is classified as superhydrophobic, which is also known as the lotus leaf effect. This principle is described by Wenzel and Cassie-Baxter’s theory [8]. The apparent contact angle $\theta^{*}$ can be expressed as follows:

(2) $$\cos\theta^{*}=f\left(1+\cos\theta \right)-1,$$

where θ is Young’s contact angle at a flat surface and f is the fraction of the area of the solid in contact with liquid. Superhydrophobic surfaces exhibit extreme water repulsion, which stems from the micro/nanostructure of the surface [9,10,11,12,13,14,15,16,17]. Such surfaces can give rise to self-cleaning, anti-frosting, and anti-corrosion properties, which renders them attractive for applied materials research [5,6,15,18,19,20]. One area where superhydrophobic surfaces show particular promise is in heat exchange systems, such as power plant condensers, where they can facilitate improved condensation heat exchange performance by inducing dropwise condensation [21,22,23,24,25,26,27,28,29]. For example, Paxson et al. reported heat transfer enhancement caused by the stable dropwise condensation initiated by the chemical vapor deposition of hydrophobic polymers, with the efficiency of the dropwise condensation process improved by using polymer surfaces exhibiting nanoscale roughness [22].

Condensation heat exchange occurs in environments that are in a supersaturated state (*S* = *P*_vapor_/*P*_wall_) in which the water vapor pressure is substantially higher than typical levels. When the degree of supersaturation increases, the superhydrophobicity becomes unstable, causing any improvements to the condensation heat exchange performance to be lost [30,31,32,33]. In order to address this issue, several studies have explored techniques to control the degree of supersaturation so that superhydrophobicity can be maintained [34,35,36,37,38,39,40,41,42,43]. 

Jo et al. conducted a study to determine the degradation parameters that control the condensation heat transfer performance of superhydrophobic surfaces with micro/nanostructures [38]. The authors defined the concept of critical gap size as the dominant criterion for the degradation of condensation heat transfer performance, with additional factors such as temperature, saturation pressure, and vapor pressure also affecting the degradation. They concluded that a smaller critical gap size increased the difficulty for fine water droplets to penetrate the surface, thereby preventing the condensation process from degrading the heat transfer performance. In a separate study, Ji et al. fabricated a replica of an actual condenser environment and compared the condensation heat transfer performance for various supersaturation conditions using a condenser tube with a superhydrophobic surface. Their experiments proved that there is a range of supersaturation in which the condensation heat transfer performance can be improved by applying a superhydrophobic surface [39]. Furthermore, in a previous study, our research team defined the concept of the droplet behavior (DB) map and proposed a simple experimental method from which the supersaturation condition is inferred according to the difference between the surface temperature and the droplet temperature. Moreover, we proposed a method for finding the conditions necessary to maintain the improved condensation performance [44].

In this study, a superhydrophobic surface with various nanostructures was fabricated using titanium, which is widely used as the main material for condensers owing to its high physical/chemical robustness. Using DB map experiments, the surface structure that would be more advantageous in improving condensation heat transfer performance was determined and the critical operating conditions were also derived. In addition, the validity of the DB map experiment was demonstrated by confirming the influence of the critical gap size on the change in the condensation performance highlighted by the DB map experiments. Our results have the potential to dramatically expand the industrial application range of heat exchange performance enhancement technology, which, at present, is restricted to a few existing materials.

## 2. Materials and Methods

### 2.1. Materials

Titanium plates (purity 99.4%, grade 1; POSCO, Pohang-si, Korea) with a dimension of 20 mm × 20 mm × 0.5 mm were used as substrates. Sulfuric acid (H_2_SO_4_, 70% concentrated solution), ethylene glycol (99.5%), ammonium fluoride (NH_4_F, >95.0%, neutral), and n-hexane (96%) were purchased from SAMCHUN Chemical, Korea. Perfluorooctyltrichlorosilane (PFOTS) was purchased from Alfa Aesar, Haverhill, MA, USA. Prior to fabricating the surface structures, impurities were removed from the surface of the titanium plates via acetone sonication.

### 2.2. Surface Preparation and Characterization

A superhydrophobic titanium surface was fabricated [10]. In order to compare the effect of surface structure, three types of titanium plates with different surface structures were manufactured. The pre-cleaned titanium plates were immersed for 24 h in 70% H_2_SO_4_ solutions maintained at 25 °C and 40 °C to produce mild etched and hard etched surfaces, respectively. During the etching reaction, the temperature of the acid solutions was regulated at 40 °C by a circulator (Lab Companion, RW-0525G). After etching, each surface was sonicated in deionized (DI) water and dried in an oven maintained at 60 °C. After being hard etched, the topmost titanium surface was anodized. An ethylene glycol mixture solution containing 1 vol% DI water and 0.25 wt% NH_4_F was used as the electrolyte, with the anodization conducted at 30 V for 4 h. Post anodization, the titanium samples were sonicated in DI water and dried in an oven for a minimum of 20 min at 60 °C. Each titanium substrate was then fluorinated via immersion in an n-hexane solution with 0.1 vol% PFOTS for 10 min. Samples were then dried in an oven for a minimum of 20 min at 60 °C. Since the nanohole structure produced by the anodization of titanium had narrow and deep holes, the resulting aspect ratio was high. Therefore, it required at least 20 min of washing and 20 min of drying to remove the electrolytes and to completely remove the water in the holes, respectively. 

The fabricated surfaces were observed using high-resolution field-emission scanning electron microscopy (SEM; JEOL, Tokyo, Japan). In order to evaluate the water-repellency of the fabricated surfaces, the contact angle and sliding angle of each surface were measured with 5 µL of droplets and by using a measuring device (Smart Drop, Femtofab, Seongnam-si, Korea). The measurements were carried out five times and the obtained values were averaged. 

### 2.3. Droplet-Behavior Experiments

**Figure 1:**
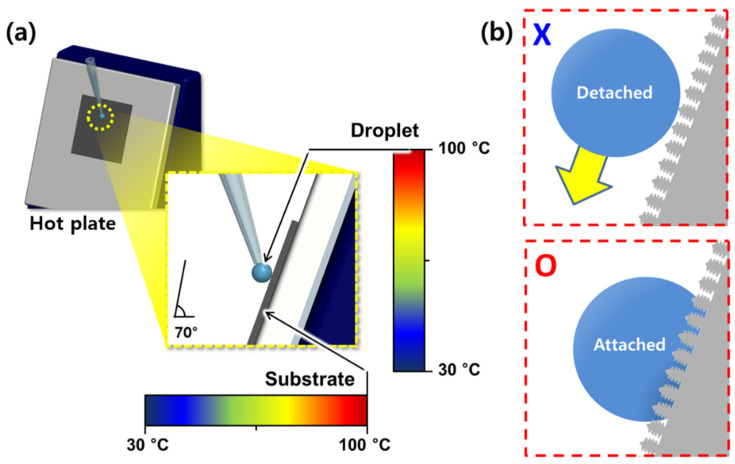
(**a**) Experimental setup for obtaining DB maps. (**b**) Illustrative diagrams of detached (X) and attached (O) droplets on a fluorinated micro/nanostructured titanium surface.

Droplet behavior experiments were conducted to investigate whether the droplet detached or attached according to the surface temperature, which is the temperature of the plate, and the droplet temperature, which is the temperature of the water droplets falling on the plate. In order to observe the droplet detachment phenomenon, the superhydrophobic sample was fixed on a hot plate designed to have an inclination of 70°. In order to increase the accuracy of the surface temperature, a K-type thermocouple having an error of ± 0.5 °C was attached on the hot plate using aluminum tape. The droplet temperature was also finely adjusted by regulating the water temperature using a hot plate and a K-type thermocouple and 10 μL of water droplets were delivered without delay using a micropipette. The setup for the droplet behavior experiments is illustrated in Figure 1. The tendency of droplets to attach to the surfaces was investigated for a fixed surface temperature while increasing the droplet temperature. The surface and droplet temperatures were adjusted from 30 to 100 °C in 5 °C intervals (Figure 1a). Droplets that adhered to the surfaces were recorded as ‘O’, while droplets that failed to adhere were labeled as ‘X’ (Figure 1b). For each condition, experiments were repeated five times to ensure the reliability of the results. The plotted ‘O’ and ‘X’ markers formed a DB map [44]. Experiments were conducted on the prepared superhydrophobic titanium surfaces to analyze the DB maps. In addition, an experiment was conducted to confirm the repeatability of the results under the same test conditions.

## 3. Results and Discussion

### 3.1. Fabrication of Superhydrophobic Titanium Surfaces

**Figure 2:**
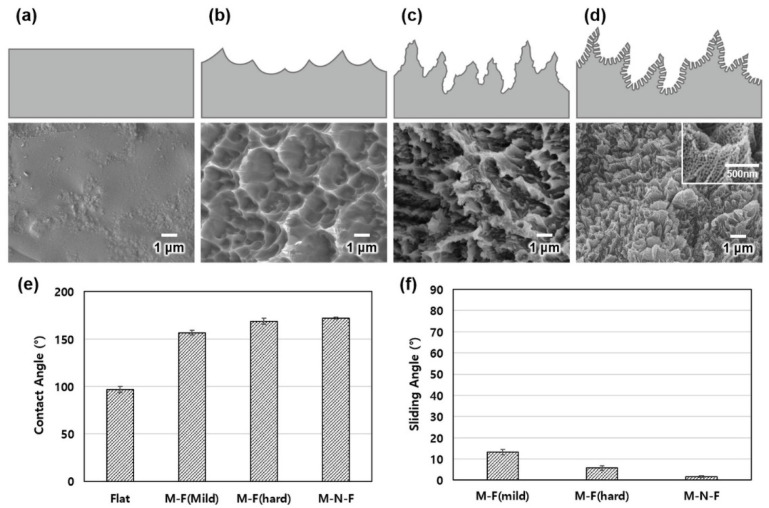
Cross-sectional diagrams and SEM images of the (**a**) original Ti, (**b**) mild-etched Ti M-F, (**c**) hard-etched Ti M-F, and (**d**) Ti M-N-F surfaces. (**e**) Contact and (**f**) sliding angles for the different Ti surfaces.

Superhydrophobic titanium surfaces were fabricated to examine the feasibility of enhancing the condensation heat transfer efficiency by modifying the superhydrophobic wettability of titanium surfaces. Furthermore, different surface structures were fabricated and DB map experiments were carried out to examine their effect on the condensation heat transfer performance. Figure 2 presents cross-sectional diagrams and SEM images of the fabricated titanium surfaces. When the flat titanium surface is exposed to H_2_SO_4_, the resulting chemical reaction causes etching and results in the formation of titanium sulfate. The etching process ends when a certain amount of etched titanium sulfate is deposited on the surface. The surface roughness caused by the etching process varies depending on the temperature of the etching solution. Etching performed at 40 °C is more intense than at 25 °C, resulting in greater surface roughness (Figure 2b,c). Following anodization, nanohole structures are formed on the titanium surfaces, which are more complex if the anodization is performed at 40 °C (Figure 2d). Although each of the surface structures were not individually fabricated, the overall surface morphology was found to be uniform. Therefore, it was possible to determine the gap size by using measurements of the images obtained by using SEM analysis. The recorded gap size was obtained by measuring the distance between peaks at 10 random locations on the sample surface and subsequently averaging these values. The gap size exhibited by the mild-etched microstructure-fluorinated (M-F) titanium surface etched at 25 °C in H_2_SO_4_ was 1.56 ± 0.22 µm, while the corresponding value for the hard-etched M-F titanium surface etched at 40 °C in H_2_SO_4_ was 0.69 ± 0.07 µm. The results show that the hard-etched M-F surface exhibited a smaller gap size than that of the mild-etched M-F. Since the micro/nanostructure-fluorinated (M-N-F) titanium surface exhibited a hierarchical structure, the gap size was measured by dividing it into two scales. At the microscale, the value was 0.63 ± 0.11 μm, which was similar to that exhibited by the hard-etched M-F surface; however, analysis at the nanoscale revealed a significantly smaller gap size of 27 ± 2 nm.

Average contact angles of 96.6° ± 6.4°, 156.8° ± 5.1°, 166.9° ± 6.5°, and 172° ± 2.4° were obtained for original, mild-etched M-F, hard-etched M-F, and M-N-F titanium surfaces, respectively (Figure 2e). As the contact angles of all the modified titanium surfaces exceed 150°, each of these surfaces can be considered superhydrophobic. In addition, the average sliding angles (Figure 2f), which represent the readiness of droplets to slide off a surface, were below 15° for all the modified surfaces, which is much lower than the 70° used in the DB map experiment. The surface inclination was fixed in order to accurately determine the effect of the variation of the surface temperature and the droplet temperature. An angle of 70° was found to be the optimum angle to observe droplet attachment and detachment.

### 3.2. Repellency Performance Evaluation via DB Mapping

When both water droplet and the superhydrophobic aluminum surface are at room temperature (around 25 °C), it is difficult for water droplets to stay on the surface even at a very small angle. Thus, on a surface tilted at an angle of 70° similar to the droplet behavior experiment, the water droplets always fall off neatly. Nevertheless, when the droplet temperature is much larger than the surface temperature, the droplets are able to adhere to the surface. The tendency of water droplets to attach/detach from surfaces based on the temperature difference between the water droplets and the surfaces can be explained by supersaturation, which is characterized as the difference in water vapor pressure. Owing to the difference between the saturated water vapor pressure at the surface temperature of the substrate (*P*_wall_) and the water vapor pressure at the surface temperature of the droplet (*P*_vapor_), a high degree of supersaturation (*S*) forms at the droplet–surface interface. Specifically, *S* represents the surface-temperature-dependent ratio of the actual and saturated water vapor pressures and it has been proposed as a metric for evaluating the condensation phenomenon [36,39,44]. It is expressed as the following.

(3) $$S=\frac{P_{vapor}}{P_{wall}}.$$

High water vapor pressure at the droplet surface causes moisture to penetrate between the nanoscale structures covering the substrate surface, resulting in the surface becoming wet. For the superhydrophobic surface with a micro/nanoscale surface structure, the attachment and detachment of droplets were tested by varying the surface and droplet temperatures from 30 °C to 100 °C.

**Figure 3:**
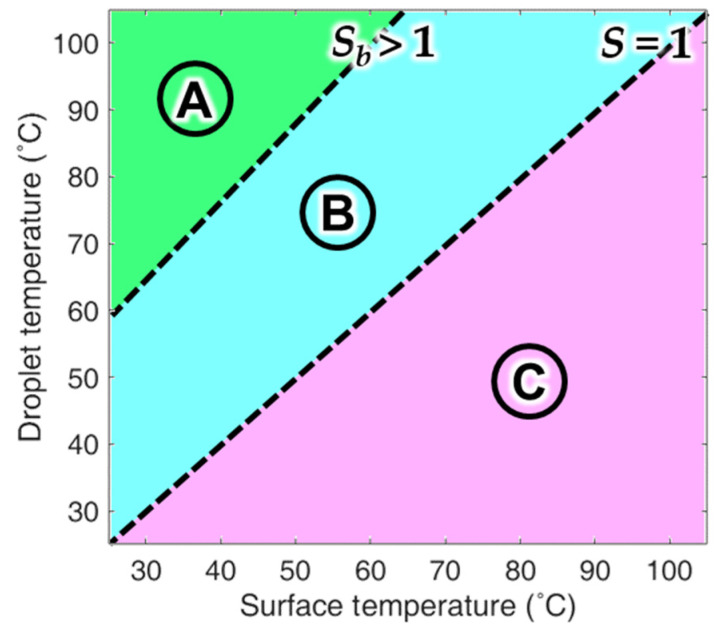
DB map showing the zones divided by boundary lines.

Figure 3 shows an example of a DB map that is divided into three zones based on the supersaturation degree and the critical temperature boundary at which the droplets are attached [44]. In theory, condensation can occur in an environment with *S* > 1; therefore, condensation does not occur naturally in zone C in Figure 3. By contrast, condensation occurs in zones A and B, where *S* > 1. Meanwhile, S_b_ refers to the boundary line between zones A and B, where *S*_b_ > 1. Based on *S*_b_, dropwise condensation occurs in zone B (where *S*_b_ *> S* > 1) and attached condensation occurs in zone A (where *S* > *S*_b_). In a previous study, DB mapping was performed with superhydrophobic aluminum. The superhydrophobic aluminum tube was manufactured and tested in an actual condenser environment and it showed an average improvement of 105% in terms of heat exchange performance in zone B. However, under the conditions in zone A, attached condensation was observed and the performance was reduced by 20% compared to a typical aluminum tube. Additionally, the surface that had undergone attached condensation in zone A did not recover its performance even when it was subsequently subjected to the conditions in zone B. It only showed the original improved condensation performance after it completely dried.

**Figure 4:**
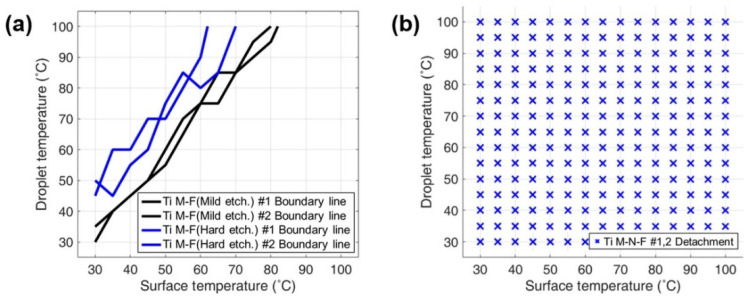
(**a**) Boundary lines of the DB maps of mild-etched Ti #1 and #2 (black lines) and hard etched Ti #1 and #2 (blue lines) surfaces. (**b**) DB map of Ti M-N-F #1, #2 surfaces.

Two samples were fabricated for each of the surface structures investigated in this study, with Figure 4 showing the DB mapping results for each surface. The DB maps corresponding to mild etched and hard etched M-F surfaces demonstrate that the boundary line of the hard-etched M-F surface, which possesses higher structural complexity and a smaller gap size, is observed at a higher value in comparison to the mild-etched M-F.

This implies that the condensation heat transfer performance can be enhanced by realizing dropwise condensation in conditions with higher supersaturation levels. The average supersaturation level across all boundary lines was found to be 1.45 and 2.5 for the mild- (*S*_bm_) and hard-etched (*S*_bh_) M-F surfaces, respectively. Therefore, in order to enhance the condensation heat transfer performance, a condensation heat exchange environment that satisfies both *S*_bm_ < 1.45 and *S*_bh_ < 2.5 must be created. As shown in Figure 4b, in the 30 °C to 100 °C temperature range, water droplets do not adhere to all sections of the M-N-F surface that contains 20 nm holes. Based on the supersaturation zone that can be inferred from the DB mapping, attached condensation will not occur even in an environment exhibiting a minimum *S* of 23. The same result was obtained upon performing DB mapping after immersing the titanium samples in water at room temperature for five weeks. The critical value of supersaturation that induces attached condensation could not be determined from this result. However, improved heat transfer performance can be achieved during operation under any oversaturated-vapor environment provided that seawater (at room temperature) is used within the condenser tubes of the actual power plant and the temperature of the steam in contact with the tube is maintained between 30 °C and 80 °C. Enhancing the condensation heat transfer performance of titanium tubes via the proposed technique is expected to provide significant economic benefits for many industrial applications.

## 4. Conclusions

In this study, we used DB mapping to estimate the environmental conditions in which the condensation heat exchange performance can be improved by using superhydrophobic titanium surfaces with chemically etched micro/nanoscale surface modifications. Additionally, we showed that the behavior of high-temperature droplets changes according to the complexity of the surface structure. Three types of superhydrophobic titanium surfaces (each with a different surface structure) were fabricated. The DB maps were constructed based on droplet behavior experiments in which the temperatures of the titanium surface and the water droplets varied. Each of the etched superhydrophobic titanium surfaces exhibited a contact angle greater than 150°. However, DB mapping revealed that the water repellency of the surfaces decreased as the droplet temperature increased at certain temperature conditions. Moreover, it was found that the water repellency improved as the size of the surface structures became smaller and more complex. As the boundary lines representing droplet attachment/detachment in the DB maps correspond to supersaturation, we found that more complex surface structures exhibited higher water-repellency stability under higher supersaturation conditions. Based on these results, it is recommended that superhydrophobic surfaces used in actual plant condensers exhibit complex surface structures. As titanium is the most widely used material in condensers in power plants, titanium was used in this study to increase the applicability of our results. Moreover, as the process can be applied regardless of the size and shape of materials, it has the potential to be used in actual mass production. Finally, following the approach outlined in this study, the application of superhydrophobic titanium surfaces is expected to improve the condensation heat transfer performance in all supersaturation zones encountered in industrial condensers.

## Data Availability

Data sharing is not applicable to this article.
